# Configurational Stability and Mobilizable Oil Release Behavior of a Multiscale Gel–Particle Cooperative Nested System in Tight Sandstone

**DOI:** 10.3390/gels12030237

**Published:** 2026-03-12

**Authors:** Baoli Liu, Bin Lü, Yishun Wang, Xiaohui Wang, Changwu Zhan, Gang Chen

**Affiliations:** 1 Second Gas Production Plant of Yanchang Gas Field, Shaanxi Yanchang Petroleum (Group) Co., Ltd., Yulin 718500, China; 2 Qingyang Exploration and Development Branch, Liaohe Oilfield Company, Yan’an 716200, China; 3 Exploration and Development Research Institute, Liaohe Oilfield Company, Panjin 124000, China; 4 Engineering Research Center of Oil and Gas Field Chemistry of Shaanxi Universities, Xi’an Shiyou University, Xi’an 710065, China

**Keywords:** multiscale gel–particle system, configurational stability, tight sandstone, interfacial regulation, mobilizable oil release

## Abstract

The configurational stability and mobilizable oil release behavior of a multiscale gel–particle cooperative nested system within tight sandstone pore structures were systematically investigated. Scanning electron microscopy (SEM), atomic force microscopy (AFM), and μCT-based three-dimensional reconstruction were employed to characterize the multiscale structural features of the system. Interfacial regulation behavior was analyzed using contact angle measurements, oil–water interfacial tension (IFT), and zeta potential tests, while core flooding experiments were conducted to evaluate seepage response and oil displacement performance. The results indicate that particle reinforcement transforms the gel pore walls from a weakly rough interface into a strongly rough and mechanically interlocked structure, with the root-mean-square surface roughness increasing from 23.6 nm to 71.4 nm. μCT quantitative analysis shows that the pore volume fraction increases from 38.6% to 52.4%, and the connectivity ratio rises from 41.2% to 68.5, leading to the formation of a more continuous pore–throat network. Interfacial property measurements reveal that the rock surface contact angle decreases from 116.3° to 60.5°, and the oil–water interfacial tension is reduced from 27 mN·m^−1^ to 3–5 mN·m^−1^. Meanwhile, the system–rock interface exhibits a stronger overall negative surface charge. During displacement experiments, the pressure differential at 3.0 pore volumes (PV) is only 17.0 kPa, significantly lower than that of the control gel (26.2 kPa). The oil recovery is increased to 44.8%, while the residual oil saturation decreases from 0.46 to 0.32, and the displacement efficiency improves from 36.1% to 55.6%. These results demonstrate that the multiscale gel–particle cooperative nested system establishes a stable, regulated seepage configuration in tight sandstone and enables sustained mobilization of trapped oil under relatively low-pressure gradients through the coupled regulation of wettability, interfacial tension, and interfacial electrostatics. This study elucidates a coupled mechanism of configurational stability–flow channel redistribution–continuous oil mobilization and provides a new material design and regulation strategy for efficient recovery of residual oil in tight reservoirs.

## 1. Introduction

Tight sandstone reservoirs are characterized by low porosity, ultra-low permeability, strong heterogeneity, and highly complex pore–throat structures. Their seepage behavior is strongly governed by a limited number of dominant flow channels, exhibiting pronounced path concentration and flow sensitivity. In such reservoirs, fluid transport is commonly constrained by micro- to nanoscale pore–throat networks, resulting in restricted sweep efficiency and dispersed residual oil that is difficult to mobilize effectively [1,2,3]. Numerous studies have demonstrated that the pore system of tight sandstone is dominated by micro- and mesopores, while the pore–throat network exhibits distinct fractal and multiscale hierarchical features. Its connectivity relies heavily on a small number of high-permeability pathways, leading to concentrated flow routes and strong sensitivity to chemical flooding and profile control systems [4,5,6,7,8]. However, most existing investigations primarily describe intrinsic pore characteristics, while quantitative understanding of how chemical agents actively reshape pore configurations and regulate flow pathways remains limited.

In recent years, research has gradually shifted toward the cooperative “structure–interface–flow” framework [9,10,11,12,13]. Mercury intrusion, low-temperature nitrogen adsorption, and X-ray micro-/nano-CT techniques have revealed pore–throat size distributions, connectivity evolution, and structural responses under pressure and salinity perturbations. Zhang et al. [14] established quantitative relationships between structural parameters and permeability in tight sandstones from the Yanchang Formation, while Liu et al. [15] emphasized the importance of three-dimensional pore–throat connectivity in predicting seepage behavior. Despite these advances, most studies focus on natural pore systems, and direct evidence for how chemical agents modify flow channel allocation and residual oil mobilization remains insufficient.

With respect to reservoir conformance control materials, polymer microspheres and particle gels (PPG) have achieved practical success in low-permeability reservoirs [16]. International studies have further evaluated regulatory behaviors of PPG in fractured tight sandstones and highlighted the necessity of controllable channel regulation strategies [17,18]. Meanwhile, nanoparticle-assisted systems have demonstrated enhanced structural reinforcement and interfacial activity [19,20,21,22], forming a parallel optimization route combining “structural regulation + interfacial modification.” Recent state-of-the-art reviews further emphasize that nanoparticles not only improve thermal and salinity resistance but also fundamentally alter pore-scale interfacial physics and transport pathways, playing a decisive role in wettability alteration, interfacial tension reduction, and flow redistribution under complex reservoir conditions [23]. In particular, the integration of nanoparticles with polymer gels or microspheres has emerged as a promising strategy for achieving multiscale structural reinforcement and dynamic channel regulation [24]. Experimental micromodel and core-scale investigations have demonstrated that morphology-tailored nanomaterials can induce sustained flow redirection and enhance oil mobilization beyond simple pressure-driven displacement mechanisms [25]. Nevertheless, most existing work relies primarily on macroscopic performance indicators, whereas systematic characterization of pore-scale configurational stability, nesting features, and channel redistribution mechanisms in realistic tight sandstone networks remains scarce.

Despite the promising performance of nanocomposite gel systems in stability and interfacial regulation, existing studies still predominantly focus on intrinsic material properties or macroscopic recovery responses. A unified mechanistic framework linking seepage configurational stability to mobilizable oil release behavior has not yet been established, resulting in a lack of traceable mechanistic connections between “material structural optimization” and “flow behavior improvement” [26,27,28].

Therefore, it is necessary to adopt a multiscale perspective integrating material structure, pore–throat configuration, and seepage response into a unified analytical framework. Accordingly, this study systematically investigates the configurational stability and displacement response of a multiscale gel–particle cooperative nested system in tight sandstone. SEM, AFM, and X-ray micro-/nano-CT techniques are employed to characterize multiscale structural features and pore–throat network configurations. Interfacial properties are evaluated through contact angle, interfacial tension, and zeta potential measurements, and core flooding experiments combined with pressure differential–permeability analysis are conducted to elucidate configurational evolution during seepage. By integrating structural, interfacial, and hydraulic responses, this work aims to bridge the gap between material design and sustained mobilizable oil release in tight sandstone reservoirs.

## 2. Results and Discussion

### 2.1. Structural and Performance Characterization of the Gels

#### 2.1.1. Microstructure and Morphology

**Figure 1:**
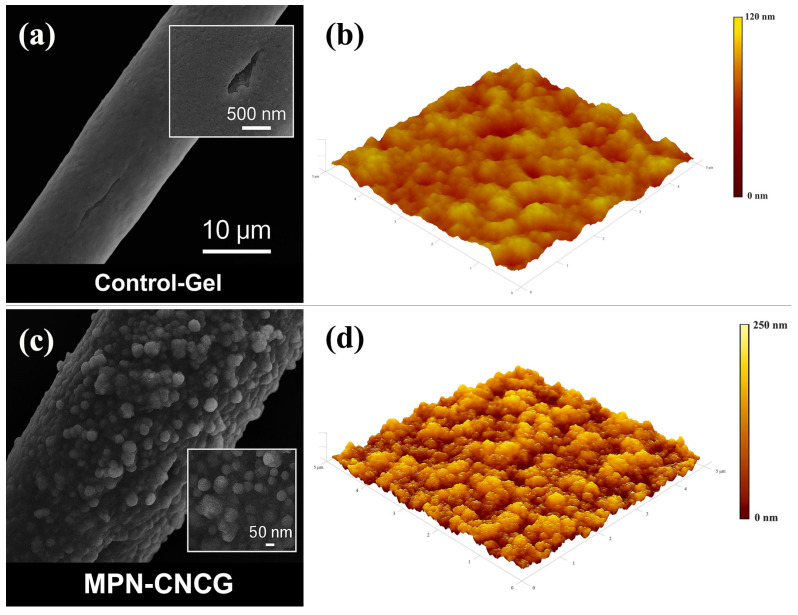
Microstructural and nanoscale roughness characterization of Control-Gel and MPN-CNCG. (**a**) SEM image of Control-Gel (×10,000), showing a relatively smooth surface with only localized microcracks; (**b**) AFM three-dimensional height map of Control-Gel (scan area: 5 × 5 μm^2^), exhibiting low surface roughness; (**c**) SEM image of MPN-CNCG (×30,000), revealing uniformly distributed nanoparticle cluster structures; (**d**) AFM three-dimensional height map of MPN-CNCG (scan area: 5 × 5 μm^2^), showing pronounced nanoscale undulations and markedly increased roughness.

The micro- to nanoscale pore-wall morphology and nanotexture of the gel samples were characterized by scanning electron microscopy (SEM) and atomic force microscopy (AFM). The detailed testing procedures and data processing methods are described in Section 4.4.1 and Section 4.4.2. The corresponding results are summarized in Table 1 and Figure 1.

The SEM observations in Figure 1 indicate pronounced differences in the microstructural features of the two gel systems. The surface of Control-Gel is mainly composed of slightly collapsed fibrous frameworks. At a field of view of 10 μm, the fibrous contours remain largely intact; however, the surface appears relatively smooth, with only a limited number of shallow cracks and small depressions (Figure 1a). When locally magnified to 500 nm, the pore-wall surface is dominated by gentle undulations and narrow grooves, without distinct nanoparticle features or protrusions. This morphology suggests that the control gel possesses a limited crosslinking density and surface construction degree and can therefore be characterized as a weakly rough and weakly anchored interface.

In contrast, the MPN-CNCG system exhibits fuller fibrous contours and a more continuous coating layer within the same observation window (Figure 1b). Dense particulate protrusions are attached to the fiber surfaces, and multiple particle clusters are locally interconnected to form bridge-like or chain-island structures. At a magnification of 500 nm, the pore walls are covered by abundant nanoscale asperities, with evident overlapping and interlacing among adjacent particles, resulting in a multiscale rough interface. These features demonstrate that the synergistic interaction between MPN and CNC markedly enhances the structural integrity and microstructural construction degree of the pore walls. In this context, the SEM-resolved pore-wall reinforcement can be regarded as the microscale structural origin of configurational stability under flow disturbance.

To further quantify the nanoscale surface differences between the two systems, AFM was employed to obtain three-dimensional topographic maps and to calculate the root-mean-square (RMS) roughness. The AFM image of Control-Gel shows that within a scan area of 5 × 5 μm^2^, the surface exhibits only minor height fluctuations, with elevations mainly distributed in the range of 0–120 nm and only a few isolated gentle protrusions (Figure 1c). The corresponding RMS roughness is 23.6 nm (Table 1), which is consistent with the SEM-observed morphology characterized by shallow grooves and sparse depressions. This indicates that the pore-wall surface of the control system provides only limited microscale anchoring sites and interfacial modulation capacity.

By comparison, the AFM topography of MPN-CNCG displays markedly enhanced peak–valley undulations, with the surface densely populated by continuously distributed particulate “gel islands” and an expanded height range of 0–250 nm (Figure 1d). Under the same scanning scale, the RMS roughness of MPN-CNCG reaches 71.4 nm (Table 1), approximately three times that of Control-Gel. This substantial increase demonstrates that nanoparticle reinforcement not only significantly enhances surface roughness but also generates a higher density of nanoscale protrusions and depressions. The quantitative increase in RMS roughness therefore suggests strengthened interfacial mechanical interlocking, which contributes to stabilizing the gel–particle configuration and improving its resistance to shear-induced disruption.

Taken together, the SEM and AFM results indicate that Control-Gel mainly provides a relatively smooth and mildly rough pore-wall interface, which is insufficient to form stable microscopic interlocking structures within pore throats. In contrast, MPN-CNCG constructs multiscale particle-island protrusions on the fibrous framework, transforming the pore wall from a mildly undulated” surface into a strongly rough interface with enhanced mechanical interlocking. On the one hand, the pronounced increase in nanoscale RMS roughness supplies more mechanical interlocking sites at the gel–rock interface, which is favorable for enhancing interfacial shear resistance and structural stability. On the other hand, the peak–valley architecture creates localized retention spaces that facilitate the trapping and immobilization of water films and residual oil droplets. These microstructural characteristics provide a solid basis for the subsequent discussion on oil–water redistribution and mobilizable oil release behavior.

#### 2.1.2. μCT Three-Dimensional Structure and Pore–Throat Network Characteristics

**Figure 2:**
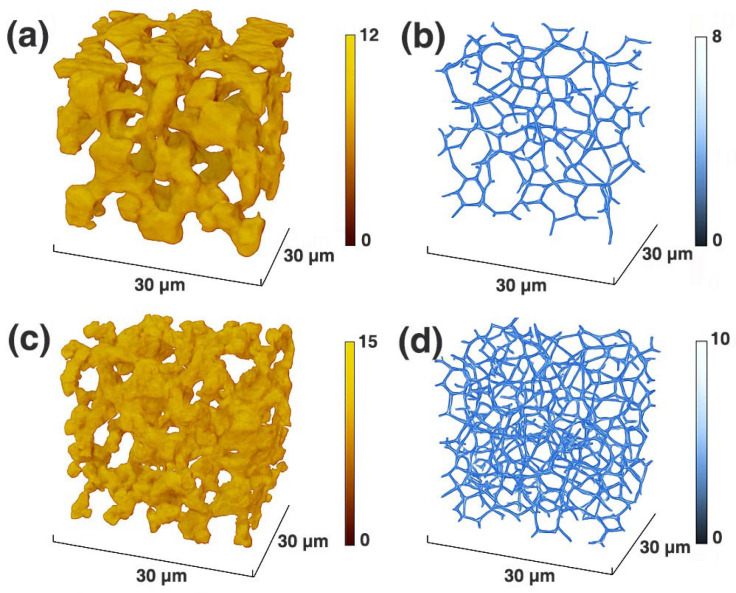
μCT-based three-dimensional reconstruction and skeletonized pore–throat networks. (**a**) Reconstructed three-dimensional pore structure of Control-Gel; (**b**) Skeletonized pore–throat network of Control-Gel; (**c**) Reconstructed three-dimensional pore structure of MPN-CNCG; (**d**) Skeletonized pore–throat network of MPN-CNCG. The reconstructed volume for all samples is 30 μm × 30 μm × 30 μm, with a voxel resolution of 2 μm/voxel.

To elucidate the construction features of the three-dimensional pore–throat network within the gels, Micro-computed tomography (μCT)-based three-dimensional reconstruction and network skeleton extraction were performed. The detailed procedures for scanning, reconstruction, and skeletonization are described in Section 4.4.3, and the corresponding results are presented in Figure 2.

Figure 2a and Figure 2c show the voxel-based three-dimensional reconstructions of the pore structures of Control-Gel and MPN-CNCG, respectively. The pore system of Control-Gel appears relatively loose, with comparatively smooth pore walls and locally distributed irregular microvoids. In contrast, due to the introduction of metal–polyphenol nanoclusters as additional crosslinking sites, MPN-CNCG exhibits a more densely interconnected pore structure in the reconstructed images. The pore-wall roughness is markedly enhanced, and the pore boundaries become more tortuous and complex.

Further skeletonization analysis (Figure 2b,d) reveals distinct topological differences between the pore–throat networks of the two gel systems. The skeleton of Control-Gel is relatively sparse, with a limited number of nodes and short connecting segments. By comparison, MPN-CNCG displays a highly branched, three-dimensional interconnected skeleton network, characterized by a significantly increased node density and a more complex and robust transport framework.

Quantitative statistics derived from the reconstructed volumes further corroborate these observations (Table 2). The average pore volume of Control-Gel is only 145.3 μm^3^, with a pore volume fraction of 38.6%, whereas these values increase to 248.7 μm^3^ and 52.4% for MPN-CNCG, respectively, indicating that nanostructured crosslinking markedly enlarges the effective pore space. The average pore–throat size increases from 5.8 μm for Control-Gel to 9.4 μm for MPN-CNCG, and the pore–throat size distribution broadens from 3–8 μm to 5–10 μm, reflecting the formation of transport channels with enhanced characteristic scales.

In addition, the node density of MPN-CNCG (543 nodes per 30 μm^3^) is 61% higher than that of Control-Gel (336 nodes per 30 μm^3^), and the average connected path length increases from 21.4 μm to 33.1 μm. As a result, the overall connectivity ratio rises significantly to 68.5%, compared with only 41.2% for Control-Gel. These results demonstrate that, under the induction of metal–polyphenol nanostructures, MPN-CNCG forms a three-dimensional pore–throat network with larger pore volumes, more nodes, longer transport paths, and higher connectivity. Such a network configuration is favorable for enhancing penetration, filling capacity, and mechanical stability within reservoir pore spaces. Notably, the simultaneous increase in connectivity and reduction in isolated domains imply that configurational stabilization is accompanied by three-dimensional network reorganization, which directly influences subsequent flow-path regulation during displacement.

#### 2.1.3. Chemical and Interfacial Property Characterization

##### Wettability Regulation Behavior

**Figure 3:**
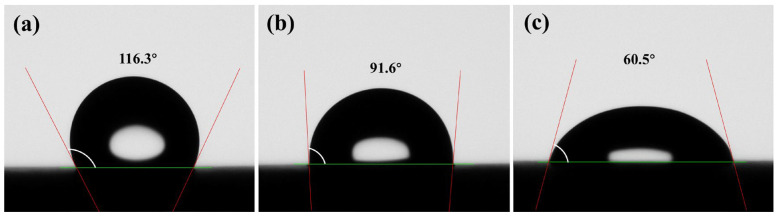
Comparison of static contact angles on rock surfaces after different treatments: (**a**) untreated rock; (**b**) Control-Gel-treated rock; (**c**) MPN-CNCG-treated rock. The red lines represent the tangent lines used for contact-angle determination, the green line indicates the solid surface baseline, and the white arc denotes the measured contact angle (θ).

To evaluate the ability of the systems to regulate rock wettability, contact angle measurements were conducted on untreated rock surfaces, Control-Gel-treated samples, and MPN-CNCG-treated samples. The detailed sample preparation and testing procedures are described in Section 4.4.4, and the experimental results are presented in Figure 3.

As shown in Figure 3, MPN-CNCG exhibits a pronounced capability to alter rock surface wettability, inducing a transition from strongly oil-wet to moderately water-wet conditions. The untreated rock surface shows a contact angle of 116.3°, characteristic of a typical oil-wet state (Figure 3a). After treatment with Control-Gel, the contact angle decreases to 91.6°, indicating a shift toward near-neutral wettability (Figure 3b). In contrast, after MPN-CNCG treatment, the contact angle is further reduced to 60.5°, demonstrating a substantial increase in surface polarity and an enhanced spreading tendency of water droplets on the mineral surface (Figure 3c). This wettability alteration is consistent with the formation of an adsorbed layer enriched in polar functional groups (e.g., carboxyl and hydroxyl groups) on the rock surface, which promotes water film stability and weakens oil–rock adhesion.

The continuous decrease in contact angle implies that the cosine term in Young’s equation (cos θ) changes from negative to positive, causing the capillary pressure term Pc = 2γcosθ/r to shift from resisting water invasion into small pores to facilitating aqueous-phase migration. Once the surface is converted from oil-wet to water-wet, the aqueous phase preferentially wets the mineral surface and forms a continuous water film along the pore walls, thereby reducing the adhesion strength of residual oil. This interfacial transition provides favorable conditions for pore–throat channel reconfiguration under a stabilized seepage configuration and subsequent mobilization of trapped oil.

In summary, the significant reduction in contact angle demonstrates that MPN-CNCG can effectively reverse wettability from oil-wet to water-wet by enhancing surface polarity. This wettability regulation establishes a critical interfacial basis for oil droplet detachment and flow channel reconnection, thereby facilitating mobilizable oil release during displacement.

##### Regulation of Oil–Water Interfacial Tension

**Figure 4:**
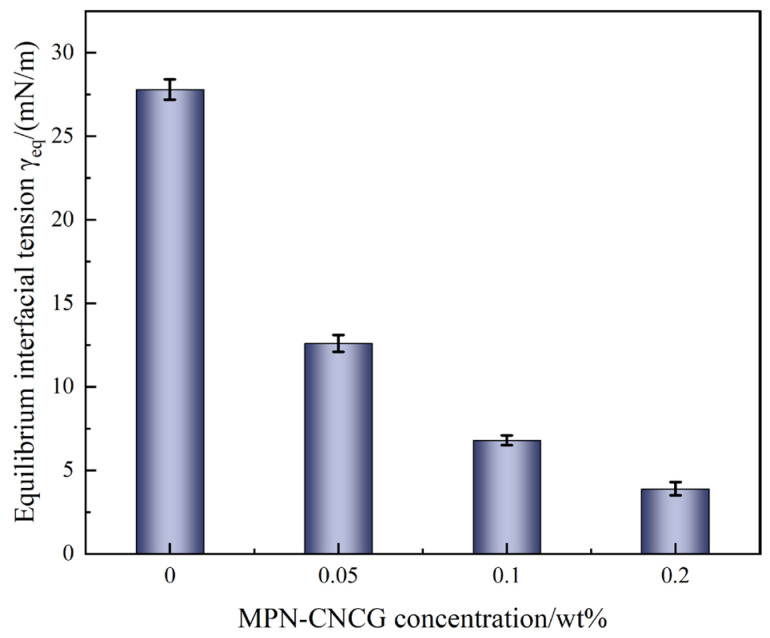
Equilibrium interfacial tension of oil–water systems at different MPN-CNCG concentrations.

**Figure 5:**
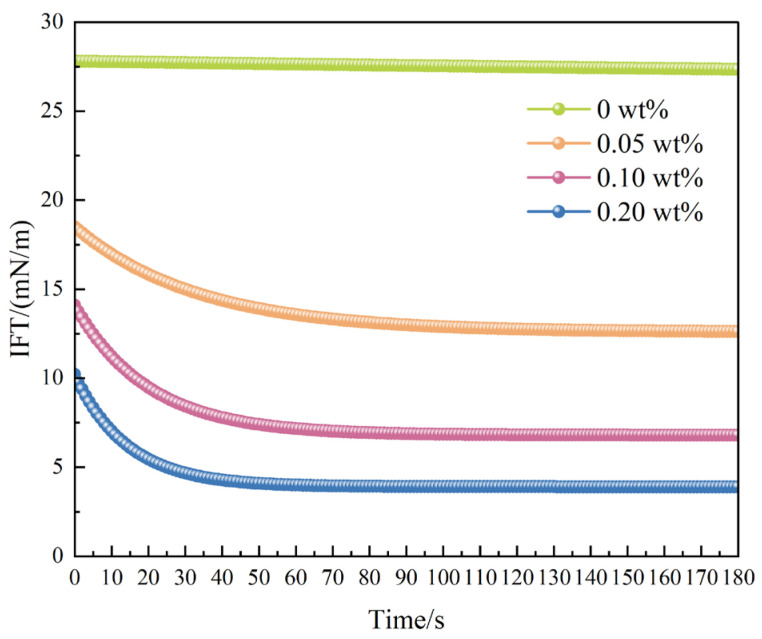
Temporal decay curves of interfacial tension (IFT).

To quantitatively characterize the effect of the system on oil–water interfacial energy, spinning drop interfacial tension (IFT) measurements were performed to determine the evolution of interfacial tension under different concentrations of MPN-CNCG. The detailed testing conditions and procedures are described in Section 4.4.5, and the results are presented in Figure 4 and Figure 5.

As shown in Figure 4, the spinning drop measurements demonstrate that MPN-CNCG markedly reduces the oil–water interfacial tension. The equilibrium interfacial tension of the blank system is 27 mN·m^−1^. Upon addition of 0.05 wt% MPN-CNCG, the interfacial tension decreases to 12.6 mN·m^−1^, and it is further reduced to 3–5 mN·m^−1^ at a concentration of 0.20 wt%, reaching the typical ultralow interfacial tension regime. The IFT–time curves in Figure 5 exhibit a rapid decay trend, indicating that the amphiphilic structural segments and nanoparticles in MPN-CNCG can quickly adsorb and reorganize at the oil–water interface. This process leads to the formation of a compact interfacial adsorption film, which substantially weakens the interfacial constraint on oil droplets.

The reduction in interfacial tension exerts a pronounced influence on oil droplet deformation, breakup, and migration. According to the Young–Laplace equation, lower interfacial tension facilitates droplet deformation and division, thereby enhancing the ability of oil droplets to pass through constricted pore throats. In addition, a reduced IFT decreases the adhesion force between oil droplets and rock surfaces, making it easier for oil to detach from pore walls and enter the flowing aqueous phase. This behavior acts synergistically with the wettability alteration revealed by the contact angle measurements, where increased water film stability and enhanced surface polarity jointly promote oil detachment and flow channel reconnection.

In summary, MPN-CNCG achieves ultralow interfacial tension through rapid interfacial adsorption and rearrangement, significantly weakening the interfacial confinement of oil droplets and promoting their deformation, detachment, and migration. This interfacial process constitutes a key driving mechanism for mobilizable oil release during displacement. In this context, the IFT reduction primarily serves to lower the interfacial energy barrier for droplet deformation and detachment, thereby enabling trapped oil to be mobilized more efficiently once the flow paths are redistributed.

##### Regulation of Interfacial Electrostatic Properties

**Figure 6:**
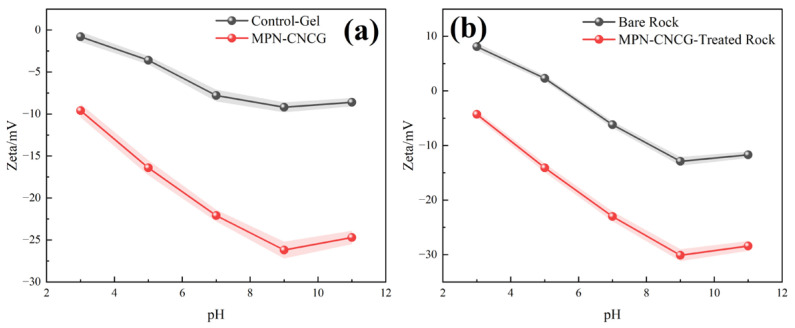
pH-dependent zeta potential characteristics of MPN-CNCG in dispersion and rock–water interface systems: (**a**) zeta potential variation in Control-Gel and MPN-CNCG dispersions with pH; (**b**) zeta potential variation in bare rock and MPN-CNCG-treated rock with pH.

To quantitatively evaluate the regulation of electrostatic properties in both the colloidal dispersion layer and the rock–water interface, pH-dependent zeta potential measurements were performed for gel dispersions and rock surface systems. The detailed sample preparation and testing procedures are described in Section 4.4.6, and the results are shown in Figure 6.

Figure 6 illustrates the zeta potential variations in gel dispersions (Control-Gel and MPN-CNCG) and rock surface systems (bare rock and MPN-CNCG-treated rock) over a pH range of 3–11. Although both systems exhibit a general trend toward more negative values with increasing pH, pronounced differences are observed in their potential ranges, variation amplitudes, and isoelectric point behaviors. These differences reflect the interfacial regulation of MPN-CNCG at both the dispersion interface and the rock–water interface levels.

For the gel dispersion systems, Control-Gel exhibits only weak negative charge over the entire pH range, with the zeta potential decreasing gradually from −0.8 mV at pH 3 to −9.2 mV at pH 9. The limited variation indicates a low density of dissociable polar groups and weak electrostatic repulsion. In contrast, MPN-CNCG shows a distinctly negative potential even under acidic conditions (−9.6 mV), and the magnitude increases rapidly with pH, reaching −26.2 mV at pH 9. This pronounced enhancement in surface charge density and electrical double-layer repulsion demonstrates that the introduction of nanoparticles and multipolar functional groups enables the formation of a stable negatively charged interfacial layer in the aqueous phase, thereby significantly improving dispersion stability and transport continuity.

Notably, the zeta potential reaches its most negative value at approximately pH 9 and then slightly increases at higher pH. This non-monotonic trend can be rationalized by the competition between surface deprotonation and electrolyte screening. Up to pH 9, progressive deprotonation of dissociable groups increases the surface negative charge density, whereas at pH > 9 the increased ionic strength associated with NaOH addition compresses the electrical double layer and partially screens surface charges, leading to a reduced absolute zeta potential. Minor contributions from pH-induced interfacial reorganization cannot be excluded.

The zeta potential response of the rock surface systems further reveals the reconstruction of mineral interfacial electrostatics induced by MPN-CNCG. Bare rock powder displays positive potential at pH 3 (+8.1 mV) and approaches its isoelectric point near pH 5, after which it gradually becomes moderately negative with increasing pH. This behavior is consistent with the typical protonation/deprotonation characteristics of hydroxyl groups on silicate mineral surfaces. By contrast, the MPN-CNCG-treated rock surface remains negatively charged across the entire pH range, with the zeta potential decreasing from −4.3 mV to −30.1 mV and an overall negative shift of approximately 15–20 mV. These results indicate that MPN-CNCG forms a stable adsorption layer on the rock surface, imparting a significantly enhanced negative charge to the originally weakly polar mineral interface and shifting the isoelectric point toward lower pH values.

From an interfacial physicochemical perspective, the overall negative shift in zeta potential implies strengthened electrical double-layer repulsion, which favors the stabilization of water films and suppresses re-adhesion of the oil phase onto rock surfaces. This electrostatic characteristic is highly consistent with the wettability alteration observed in contact angle measurements and the pronounced reduction in interfacial tension. Collectively, these effects demonstrate that MPN-CNCG provides a dual interfacial regulation pathway—enhancing the negative charge of dispersions and reconstructing the surface charge distribution of rocks—thereby establishing a robust electrochemical basis for preferential water spreading, oil droplet detachment, and mobilizable oil release.

In summary, zeta potential measurements confirm from an electrochemical standpoint the systematic regulation of interfacial charge by MPN-CNCG. The synergistic effects on dispersion stability and rock surface charge reconstruction form a critical foundation for subsequent wettability reversal, interfacial energy reduction, and improvement of pore-scale seepage behavior associated with stable configurational regulation and sustained mobilizable oil release.

Collectively, the concurrent reduction in contact angle and interfacial tension, together with the enhanced negative surface charge, defines an integrated interfacial regulation pathway governing oil droplet dynamics at the pore scale. Wettability reversal weakens oil–rock adhesion and stabilizes aqueous films along pore walls; ultralow interfacial tension lowers the capillary constraint on droplet deformation and division; meanwhile, strengthened electrostatic repulsion suppresses re-adhesion and coalescence after detachment. Under the combined action of these physicochemical factors, trapped oil droplets can be more readily detached, elastically deformed, and transported across constricted pore throats, thereby establishing the interfacial precondition for sustained mobilizable oil release within the regulated seepage configuration.

### 2.2. Seepage Configurational Stability of the Multiscale Gel–Particle System

#### 2.2.1. Pressure Differential Response During Injection and Displacement

**Figure 7:**
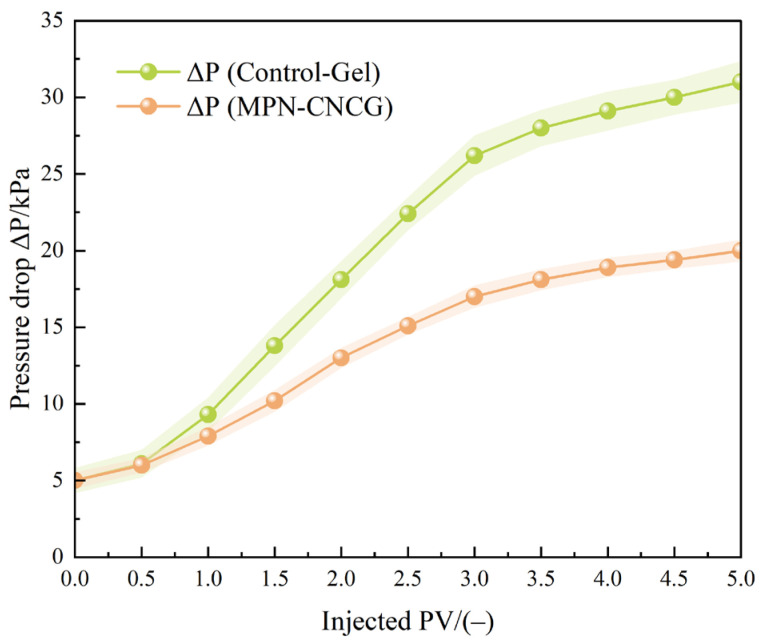
Variation in core pressure differential (ΔP) with injected pore volume (PV) during injection and displacement.

To quantitatively characterize the seepage configurational behavior of the multiscale gel–particle cooperative nested system in tight sandstone pores, the pressure differential–injected pore volume (ΔP–PV) responses of cores treated with Control-Gel and MPN-CNCG were comparatively analyzed (the experimental procedure is described in Section 4.4.7). The corresponding results are presented in Figure 7. Under identical baseline flooding conditions, the initial pressure differential for both core groups was 5.0 kPa, ensuring comparable initial states.

As shown in Figure 7, both systems induce a pronounced increase in ΔP during the injection stage; however, their growth magnitudes and evolution patterns differ markedly. For the Control-Gel system, ΔP rises to 18.1 kPa when the injected volume reaches 2.0 PV. As the injection volume increases further to the range of 3.0–5.0 PV, ΔP continues to increase to 26.2–31.0 kPa and gradually enters a high-resistance plateau. This rapid rise and sustained high-pressure response indicate that the control gel tends to form localized retention and nonuniform distributions at the pore scale, thereby strongly perturbing the original seepage pathways.

In contrast, the pressure buildup associated with the MPN-CNCG system is considerably more moderate. At an injection volume of 2.0 PV, ΔP is only 13.0 kPa, corresponding to 71.8% of that for the Control-Gel system. When the injected volume reaches 5.0 PV, ΔP stabilizes at approximately 20.0 kPa and remains consistently lower than that of the control system. Moreover, no abrupt pressure surges or strong fluctuations are observed throughout the injection process, suggesting that MPN-CNCG distributes more continuously and uniformly within the pore network.

During the subsequent post-flush with formation water, the pressure differential associated with MPN-CNCG remains within a controllable range and gradually decreases with continued flushing, without sudden drops or abnormal oscillations. This behavior indicates that the action of MPN-CNCG in the pore space does not rely on transient localized plugging but instead maintains a relatively stable configurational distribution under sustained flow conditions.

Taken together, the ΔP–PV response characteristics demonstrate that the multiscale gel–particle cooperative nested system establishes a continuous and tunable seepage configuration in tight sandstone pores. First, its pressure differential remains significantly lower than that of the conventional control gel, avoiding severe localized blockage effects. Second, the smooth evolution of ΔP reflects a more homogeneous and stable spatial distribution of the system. Such seepage configurational features provide essential hydraulic conditions for subsequent flow channel redistribution and mobilizable oil release. Accordingly, the ΔP–PV evolution is used here as a macroscopic indicator to evaluate whether the established seepage configuration remains continuous and controllable during injection and post-flushing.

#### 2.2.2. Permeability Response and Channel Regulation Under Steady-State Seepage Conditions

**Figure 8:**
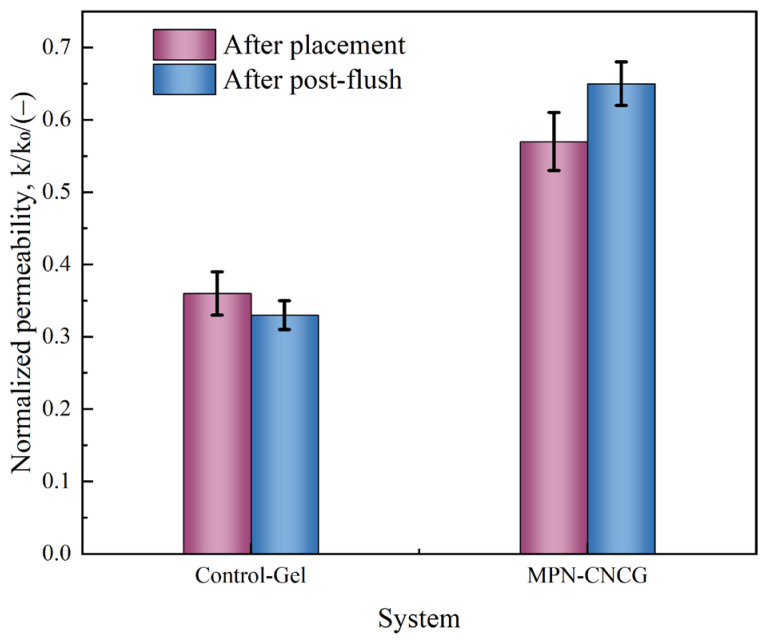
Normalized permeability (k/k_0_) of tight sandstone cores treated with Control-Gel and MPN-CNCG at different flooding stages.

To further elucidate the channel regulation behavior of the systems, the normalized permeability (k/k_0_) under different treatment conditions was comparatively analyzed (the calculation and testing procedures are described in Section 4.4.8). The corresponding results are presented in Figure 8.

As shown in Figure 8, the two systems exhibit fundamentally different modes of interaction with seepage channels. For the Control-Gel system, the core permeability decreases from its initial value to 0.36 k_0_ after gel injection and further declines to 0.33 k_0_ following post-flush with formation water. The permeability remains persistently low, indicating that the flow resistance structures formed by the control gel within the pore space are largely irreversible. This behavior suggests that the control gel primarily acts by directly occupying and locally sealing the original dominant flow channels.

In contrast, the MPN-CNCG system shows a distinctly different permeability response. After system injection, the core permeability is 0.57 k_0_, which is significantly higher than that observed for the Control-Gel system. During the subsequent formation water flooding stage, the permeability further recovers to 0.65 k_0_, demonstrating a certain degree of reversibility and structural reconfiguration. These results indicate that the multiscale gel–particle system does not cause complete loss of flow pathways but instead regulates local high-permeability channels while preserving overall network connectivity.

When combined with the pressure differential response results, it can be inferred that the permeability reduction induced by Control-Gel mainly arises from direct blockage of dominant flow channels. In contrast, MPN-CNCG achieves redistribution of flow paths through cooperative multiscale embedding: while high-permeability channels are selectively suppressed, low-permeability regions remain effectively connected. This mechanism avoids excessive deterioration of the overall seepage capacity.

Therefore, from the perspective of steady-state permeability response, the multiscale gel–particle cooperative nested system establishes a flow-regulating configuration rather than an irreversible blocking state in tight sandstone. Its defining features include a controllable reduction in permeability and partial recoverability. Such a channel regulation mode provides an essential seepage structural basis for subsequent fluid redistribution and mobilizable oil release. From a functional perspective, the k/k_0_ response serves to distinguish channel regulation with preserved connectivity from irreversible blockage, thereby linking seepage configurational behavior to the effectiveness of mobilizable oil release.

#### 2.2.3. Pressure Differential Recovery and Re-Flooding Behavior

**Figure 9:**
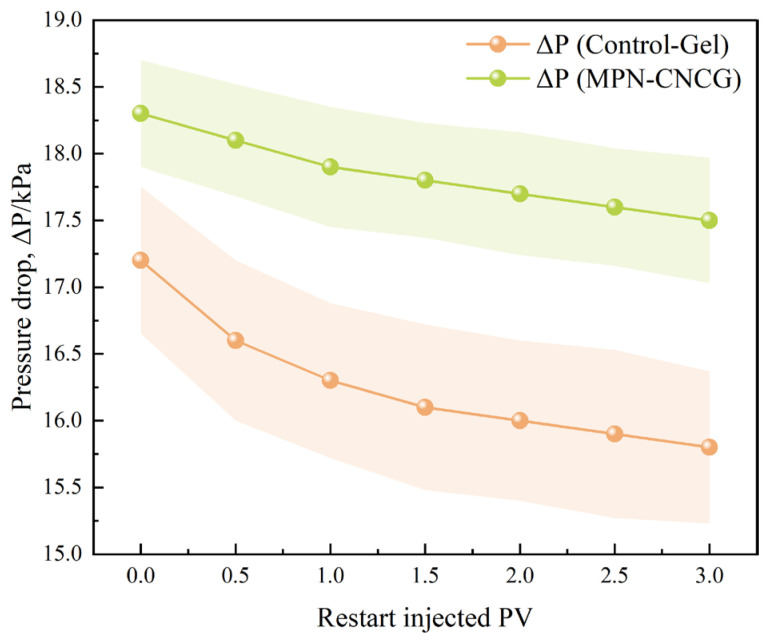
Pressure differential recovery behavior during the re-flooding stage.

To verify the configurational stability of the multiscale gel–particle cooperative nested system within pore spaces, core injection was suspended after the system reached a steady state. Flooding was then restarted using simulated formation water. The pressure differential recovery behaviors of different systems during the re-flooding stage were comparatively analyzed (Table 3 and Figure 9).

For the Control-Gel system, the pressure differential under steady-state conditions before flow interruption is 30.5 kPa. After flooding is restarted, the pressure differential decreases to 17.2 kPa and further declines to 15.8 kPa during the subsequent 3 PV post-flush. The corresponding recovery ratio R_1_ is 0.564, and the retention ratio R_2_ is 0.518, indicating that the control gel is unable to maintain its original flow resistance state during interruption and restart. This behavior suggests that the retained structures formed by the control gel within the pore space are highly sensitive to changes in flow conditions.

In contrast, the MPN-CNCG system exhibits a pressure differential of 19.8 kPa under steady-state conditions before flow interruption. After re-flooding is initiated, the pressure differential remains at 18.3 kPa and stabilizes at 17.5 kPa during the subsequent flushing process. The corresponding recovery ratio R_1_ reaches 0.924, and the retention ratio R_2_ is 0.884, demonstrating that this system can sustain a relatively high level of seepage resistance even under stop–restart conditions.

Further examination of the dynamic evolution during the re-flooding stage (Figure 9) shows that the pressure differential of the Control-Gel system continuously decays with increasing injected pore volume, whereas the MPN-CNCG system exhibits only minor variations and remains within a relatively stable range. This contrast indicates that the resistance structures formed by the control gel progressively disintegrate during re-flooding, while the multiscale gel–particle cooperative nested system preserves a continuous seepage response after flow regime switching.

Taken together, both the terminal indices and the recovery process characteristics demonstrate that the resistance structures formed by the Control-Gel system are transient in nature. In contrast, the MPN-CNCG system establishes a stable seepage configuration in tight sandstone pores. This configuration can maintain its regulatory function under interruption and restart conditions, thereby providing a persistent hydraulic basis for subsequent flow channel redistribution and mobilizable oil release. This stop–restart verification therefore specifically serves to confirm configurational stability under flow perturbation, which is a prerequisite for sustaining channel regulation rather than relying on transient plugging.

#### 2.2.4. Identification of Configurational Stability Based on Seepage Responses

Based on the seepage response characteristics, the configurational stability of the multiscale gel–particle cooperative nested system in tight sandstone pores can be systematically identified. By comparatively analyzing the pressure differential evolution during the main flooding stage (Section 2.2.1), the steady-state permeability responses (Section 2.2.2), and the pressure recovery behavior under re-flooding conditions (Section 2.2.3), the modes of interaction of different systems with pore networks and their stability can be evaluated from a macroscopic seepage perspective.

During the primary flooding stage, the MPN-CNCG system exhibits a continuous and controllable pressure differential response without abrupt plugging events. The steady-state permeability results further indicate that the treated cores retain relatively high normalized permeability values, demonstrating that the regulation of flow channels by this system differs fundamentally from the irreversible blocking structures typically formed by conventional gels. Under stop–restart flooding conditions, both the recovery ratio and retention ratio of the MPN-CNCG system remain at high levels and evolve smoothly with injected pore volume, indicating that a stable seepage response is maintained even during flow regime transitions.

By contrast, the control gel system shows more pronounced pressure decay and permeability reduction at all stages. During re-flooding, the pressure differential drops rapidly, suggesting that the resistance structures formed by the control gel are closer to transient retention or weakly stable configurations and are highly sensitive to flow perturbations.

Overall, these multidimensional seepage response characteristics provide converging evidence that the multiscale gel–particle cooperative nested system tends to establish a relatively stable flow-regulating configuration in tight sandstone pores. Rather than being primarily governed by irreversible single-point blockage, the observed permeability reversibility, smooth pressure evolution, and high recovery/retention ratios collectively suggest a dominant mode of sustained flow pathway adjustment.

It should be noted, however, that localized retention or partial structural rearrangement under flow cannot be completely excluded. Nevertheless, the consistency among pressure evolution, permeability response, and re-flooding stability indicates that configurational regulation of pore-scale channels plays a prevailing role in the overall seepage behavior.

This inference provides a mechanistic basis for subsequent analyses of fluid redistribution and mobilizable oil release from a macroscopic seepage perspective.

### 2.3. Evaluation of Mobilizable Oil Release Under Multiscale Configurational Regulation

#### 2.3.1. Enhancement of Oil Displacement Efficiency and Recovery Factor

**Figure 10:**
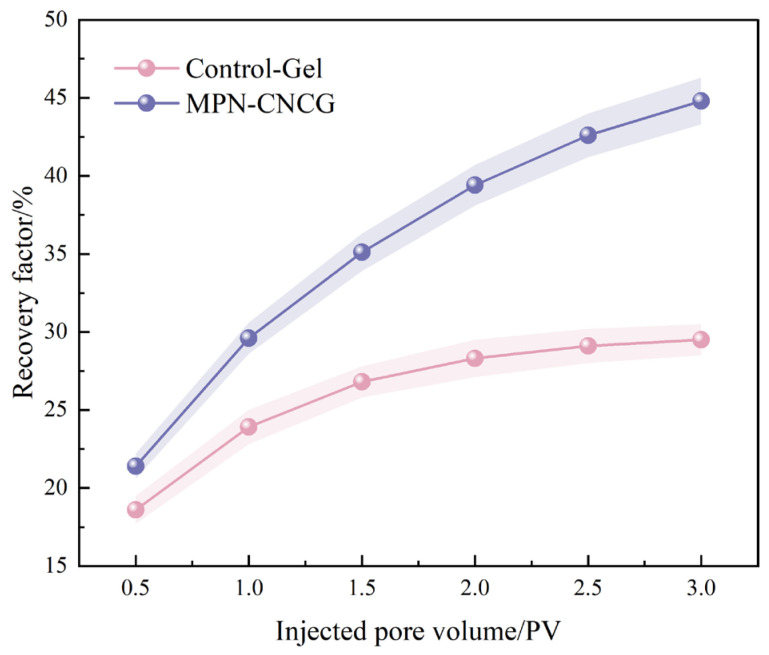
Recovery factor as a function of injected pore volume for cores treated with Control-Gel and MPN-CNCG.

To evaluate the effect of the multiscale gel–particle cooperative nested system on mobilizable oil release in tight sandstone, post-waterflooding experiments were conducted on cores treated with different systems after the establishment and stability characterization of seepage configurations (Section 2.2). The evolution of recovery factor and the corresponding oil displacement efficiency were comparatively analyzed, with the results shown in Figure 10 and Table 4.

As indicated by the recovery factor evolution with injected pore volume (Figure 10), the cores treated with the control gel exhibit a certain degree of oil production response during the early stage of displacement. However, once the injected volume exceeds 2.0 PV, the increase in recovery factor becomes markedly attenuated, and the final recovery reaches only 29.5%. In contrast, the cores treated with MPN-CNCG maintain a higher level of oil production throughout the entire displacement process, with the recovery factor continuously increasing and reaching 44.8% at 3.0 PV, which is significantly higher than that of the control system.

Further analysis of the oil displacement efficiency (Table 4) reveals pronounced differences in residual oil saturation after flooding. Based on the initial oil saturation (S_oi_) and residual oil saturation (S_or_), the calculated oil displacement efficiency (E_m_) for the control gel system is 36.1%, whereas that for the MPN-CNCG system increases to 55.6%. This result indicates that the multiscale gel–particle cooperative nested system is substantially more effective in reducing residual oil saturation within the cores.

Comparison of the macroscopic recovery behavior with the microscopic displacement efficiency suggests that oil production for the control gel system is mainly concentrated in the early flooding stage, with limited capability to mobilize the remaining oil during subsequent displacement. By contrast, the MPN-CNCG system sustains a stable oil production response over a broader range of injected pore volumes. The continuous increase in recovery factor, together with the pronounced improvement in displacement efficiency, demonstrates that this system not only enhances initial oil mobilization but also persistently promotes the participation of residual oil during later flooding stages.

These results show that the oil displacement performance of the multiscale gel–particle cooperative nested system in tight sandstone is not merely manifested as a transient enhancement of oil production. Instead, it is reflected in both a substantial increase in overall recovery factor and an effective reduction in residual oil saturation. This dual improvement provides an experimental basis for subsequent analyses of mobilizable oil release from the coupled perspective of pressure response and production behavior. Here, the recovery factor and Em are used as direct quantitative outputs of mobilizable oil release, providing macroscopic validation of the effectiveness of configurational regulation.

#### 2.3.2. Coupled Pressure Differential–Production Response During Mobilizable Oil Release

To elucidate how the multiscale gel–particle cooperative nested system regulates mobilizable oil release during displacement, the pressure differential data (Figure 7) and recovery factor data (Figure 10) obtained synchronously from the same set of core flooding experiments were reorganized and matched at identical injected pore volumes (PV). The corresponding relationships are summarized in Table 5.

Overall, both systems exhibit a common trend of increasing pressure differential and recovery factor with increasing injected pore volume. However, pronounced differences are observed in the correspondence between pressure level and production efficiency. For the Control-Gel system, as PV increases from 0.5 to 3.0, the pressure differential rises sharply from 6.1 kPa to 26.2 kPa, whereas the recovery factor increases only from 18.6% to 29.5%. The substantial pressure buildup is not accompanied by a proportional increase in oil production, indicating that this system mainly relies on elevated flow resistance to induce initial oil mobilization, while its capability for sustained release of residual oil during later flooding stages remains limited.

In contrast, the MPN-CNCG system consistently maintains a lower pressure differential under the same PV conditions but achieves significantly higher recovery factors than the control system. At 2.0 PV, the pressure differential of MPN-CNCG is 13.0 kPa, markedly lower than that of Control-Gel (18.1 kPa), whereas the recovery factor reaches 39.4%, exceeding that of the control system (28.3%). When the injected volume increases to 3.0 PV, the pressure differential of MPN-CNCG is 17.0 kPa—approximately two-thirds of that of the control system—while the recovery factor further increases to 44.8%, in sharp contrast to the 29.5% achieved by the control system.

Further comparison of the relationship between pressure increment and recovery increment over different PV intervals reveals distinct response patterns. The Control-Gel system exhibits progressively smaller gains in recovery during the stage of rapid pressure increase (1.5–3.0 PV), displaying a typical a high-pressure build-up with limited incremental recovery behavior. By contrast, the MPN-CNCG system maintains a steady increase in recovery while the pressure differential rises gradually, exhibiting a moderate pressure increase accompanied by sustained recovery growth response mode. These results demonstrate that the multiscale gel–particle cooperative nested system does not enhance oil recovery by forming highly resistive blockage states, but rather by maintaining a stable flow-regulating configuration that progressively activates additional pore volumes. Instead, it achieves effective mobilizable oil release through continuous regulation of pore-scale flow pathways, enabling progressively larger pore volumes to participate in the displacement process.

Taken together, the coupled analysis of pressure differential and production behavior indicates that the Control-Gel system forms a transient seepage state dominated by localized flow resistance, with its oil displacement performance concentrated mainly in the early flooding stage. In contrast, the MPN-CNCG system establishes a stable flow-regulating configuration that enables sustained enhancement of recovery under relatively low pressure differentials. These findings suggest that the promotion of mobilizable oil release by the multiscale gel–particle cooperative nested system originates from optimization of seepage configuration rather than from simple amplification of flow resistance, providing experimental evidence for subsequent mechanistic interpretation from a configurational regulation perspective.

#### 2.3.3. Mechanistic Role of Multiscale Configurational Stability in Mobilizable Oil Release

Based on the seepage configurational stability characterized in Section 2.2 (ΔP–PV response, normalized permeability k/k_0_, and pressure recovery/retention indices) and the quantitative evaluation of mobilizable oil release in Section 2.3.1 and Section 2.3.2 (recovery factor, oil displacement efficiency, and ΔP–recovery correspondence), a closed-loop linkage among configurational stability, channel regulation, and mobilizable oil release can be established to explain the sustained oil production achieved by the MPN-CNCG system in tight sandstone.

1. Configurational stability enables sustained regulation rather than transient plugging

During injection and displacement, MPN-CNCG exhibits a smooth and controllable pressure evolution without abrupt surges. At 2.0 PV and 3.0 PV, its pressure differentials (13.0 and 17.0 kPa) remain markedly lower than those of the control system (18.1 and 26.2 kPa), indicating the absence of strong localized blockage. Moreover, under stop–restart flooding, the pressure recovery and retention ratios of MPN-CNCG (R_1_ = 0.924, R_2_ = 0.884) are significantly higher than those of the control gel. This behavior demonstrates that MPN-CNCG maintains a stable seepage response under flow perturbation, providing a prerequisite for sustained regulation.

2. Channel regulation suppresses dominant pathways while preserving global connectivity

Steady-state permeability results further reveal distinct regulation modes. After injection, k/k_0_ decreases to 0.36 for Control-Gel and further to 0.33 after post-flushing, reflecting an irreversible blocking effect. In contrast, for MPN-CNCG, k/k_0_ is 0.57 after injection and recovers to 0.65 after post-flushing, indicating a controllable reduction with partial recovery. These results suggest that MPN-CNCG forms a regulatory seepage configuration that redistributes dominant flow channels while maintaining sufficient connectivity for continuous displacement.

3. Mobilizable oil release is achieved under lower pressure differentials

The ΔP–recovery correspondence shows that MPN-CNCG consistently attains higher recovery factors at lower pressure differentials. At 2.0 PV, it achieves 39.4% recovery at 13.0 kPa, compared with 28.3% at 18.1 kPa for the control system. At 3.0 PV, the recovery increases to 44.8% at 17.0 kPa, whereas the control system reaches only 29.5% at 26.2 kPa. This contrast indicates that the high pressure of the control system mainly reflects energy dissipation by localized blockage, while the regulatory configuration of MPN-CNCG enables displacement energy to activate larger pore volumes, leading to sustained oil release and a pronounced reduction in residual oil saturation (S_or_ = 0.32, E_m_ = 55.6%).

4. Mechanistic summary

**Figure 11:**
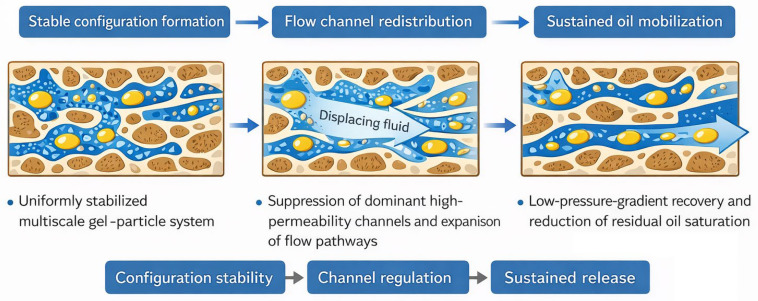
Schematic illustration of movable oil release regulated by the stable multiscale gel–particle configuration in tight sandstone. (The **left** panel shows the formation of a uniformly stabilized multiscale gel–particle system within the pore space, establishing a continuous and stable configuration. The **middle** panel illustrates the redistribution of flow channels caused by the suppression of dominant high-permeability pathways and the expansion of alternative flow routes under fluid displacement. The **right** panel depicts the sustained mobilization of trapped oil under a reduced pressure gradient, leading to an increase in recovery factor and a decrease in residual oil saturation. This schematic summarizes the sequential processes of configuration stability, channel regulation, and sustained oil release induced by the multiscale gel–particle system.

In summary, the enhanced oil displacement performance of MPN-CNCG does not rely on forming stronger blockage or higher pressure differentials. Instead, it originates from stable multiscale configurational regulation that weakens the dominance of single preferential channels while preserving overall connectivity. This transforms the displacement process from localized, short-term production into sustained multichannel participation, achieving higher recovery with lower pressure cost. A schematic illustration of this mechanism is provided in Figure 11.

From an engineering perspective, the multiscale gel–particle cooperative nested system differs from conventional field drive materials that primarily rely on high-strength plugging or bulk resistance buildup. Instead of forming irreversible blockage states, the present system establishes a flow-regulating configuration under moderate pressure differentials, which is particularly advantageous for tight sandstone reservoirs where excessive pressure gradients may induce formation damage or fracture propagation.

Nevertheless, it should be noted that the current evaluation was conducted under controlled laboratory conditions at ambient temperature and salinity. The long-term stability, thermal resistance, and performance of the system under high-temperature and high-salinity reservoir environments require further investigation before large-scale field application.

## 3. Conclusions

1. The multiscale gel–particle cooperative nested system establishes a stable “regulatory” seepage configuration within tight sandstone pores. This configuration is characterized by smooth pressure evolution during injection and re-flooding, as well as a controllable and partially recoverable reduction in permeability. Compared with the irreversible blocking structures formed by the control gel, this system suppresses the dominance of single high-permeability channels while preserving overall network connectivity, enabling sustained redistribution of flow pathways. These seepage responses confirm that the established configuration is both stable and tolerant to flow perturbations. Among the identified mechanisms, configurational stability at the pore–throat scale is clarified as the primary structural driver, forming the fundamental basis for subsequent channel redistribution and interfacial regulation.

2. At the pore scale, the system simultaneously regulates wettability, interfacial tension, and interfacial electrostatic properties, converting rock surfaces from oil-wet to water-wet and creating a low-energy, negatively charged interfacial environment. The reduction in contact angle and the establishment of ultralow interfacial tension weaken oil–rock adhesion, while the enhanced electrical double-layer repulsion stabilizes the water film and suppresses oil re-adhesion. Collectively, these interfacial effects lower the energy barriers for oil droplet detachment, deformation, and migration from a physicochemical perspective. In comparison with structural regulation, interfacial reconstruction functions as a synergistic enhancement mechanism rather than an independent control factor, facilitating continuous oil release once stable seepage pathways are established.

3. Through the combined action of stable configurational regulation and interfacial reconstruction, the multiscale gel–particle system achieves higher recovery factors and significantly lower residual oil saturation under relatively low pressure differentials. Its oil displacement enhancement does not rely on strong localized plugging or high energy consumption but instead arises from progressive participation of more pore channels in the displacement process, leading to sustained multichannel mobilizable oil release. These results demonstrate that configurational stability and interfacial regulation jointly govern the long-term efficiency of residual oil mobilization in tight sandstone reservoirs. The relatively low pressure differential required to achieve enhanced recovery implies reduced injection-energy demand and potential operational cost advantages under pressure-constrained field conditions. From an environmental and sustainability perspective, avoiding excessive pressure buildup and irreversible plugging may mitigate formation damage risks; however, uncertainties remain regarding long-term stability under high-temperature/high-salinity reservoir conditions, cyclic injection performance, scale-up behavior in heterogeneous formations, and the transport and environmental fate of nanoscale components. These aspects warrant further systematic investigation to ensure field applicability and environmental compatibility.

## 4. Materials and Methods

### 4.1. Materials

The materials used in this study are summarized in Table 6.

### 4.2. Experimental Instruments

The major experimental instruments and their specifications are summarized in Table 7.

### 4.3. Synthesis Procedure

**Figure 12:**
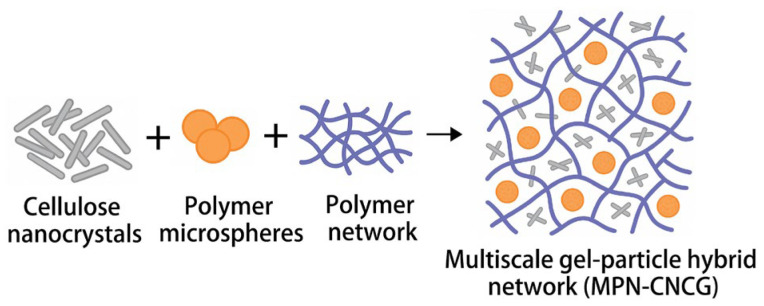
Composition, assembly process, and final multiscale nested structure of the MPN-CNCG composite.

To construct the multiscale gel–particle cooperative nested system, an in situ free-radical crosslinking strategy was adopted, in which nanoscale reinforcing units and microscale nested particles were synchronously incorporated during gel network formation to achieve cooperative multiscale structuring. A schematic illustration of the synthesis procedure is shown in Figure 12.

Specifically, 200.0 mL of deionized water was added into a 500 mL glass beaker and pre-stirred at 30 °C and 300 rpm for 5 min using a magnetic stirrer. Subsequently, acrylamide (AAm, 1.80 g), 2-acrylamido-2-methylpropane sulfonic acid (AMPS, 0.90 g), and methacryloyloxyethyl trimethyl ammonium chloride (DMC, 0.30 g) were added sequentially, maintaining the total monomer concentration at 1.5 wt%. Then, N,N′-methylenebisacrylamide (MBAA, 0.045 g, corresponding to 1.5 wt% of the total monomer mass) was introduced as the crosslinker, and the solution was stirred for an additional 10 min to ensure complete dissolution.

Cellulose nanocrystals (CNC, average particle size 80–120 nm, 0.20 g) were separately dispersed in 20 mL of deionized water and ultrasonicated at 120 W for 15 min to obtain a stable suspension. The CNC suspension was added dropwise to the monomer solution within 5 min under continuous stirring at 300 rpm and further mixed for 20 min, allowing CNC to be uniformly dispersed as nanoscale reinforcing nodes.

Pre-synthesized crosslinked polymer microspheres (average diameter 30–80 μm, in the swollen state, 1.50 g) were then introduced to achieve a mass fraction of approximately 0.7 wt%, and the mixture was stirred at 150 rpm for 10 min to ensure homogeneous distribution, thereby forming potential microscale nested units.

An ammonium persulfate (APS)–sodium bisulfite (NaHSO_3_) redox initiator system was employed, with APS and NaHSO_3_ each added at 0.10 g, corresponding to a total initiator dosage of approximately 3.3 wt% relative to the monomer mass. Immediately after initiator addition, the reaction mixture was transferred into a sealed reactor and polymerized at 45 °C in a thermostatic water bath for 2 h. During this process, zwitterionic polymer chains underwent in situ free-radical crosslinking in the presence of CNC and microspheres, progressively forming a three-dimensional gel network containing nanoscale reinforcing nodes and microscale nested structures.

After gelation, the pH of the system was adjusted to 7.2–7.5, followed by aging at room temperature for 12 h to complete network rearrangement and structural stabilization. The obtained gel was then gently extruded through a 200-mesh nylon sieve to remove free water and stored in sealed containers for subsequent use. The final product was a multiscale gel–particle cooperative nested material (MPN-CNCG), consisting of a chemically crosslinked gel network as the continuous phase with synergistically embedded cellulose nanocrystals and polymer microspheres.

### 4.4. Methods

#### 4.4.1. SEM Imaging

The samples were freeze-dried at −50 °C and sputter-coated with gold prior to observation. Scanning electron microscopy (SEM) was performed using a KYKY-EM6900 scanning electron microscope (Beijing Zhongke Instrument Co., Ltd., Beijing, China) at an accelerating voltage of 5 kV to acquire micro to nanoscale images of pore-wall morphologies. The SEM images were used to characterize the fibrous skeleton structure, particle attachment features, and differences in pore-wall compactness.

#### 4.4.2. AFM Mapping

Atomic force microscopy (AFM) measurements were conducted using a Dimension Icon atomic force microscope (Bruker Nano Surfaces Division, Beijing, China) in tapping mode to obtain two-dimensional and three-dimensional surface height maps. The scanning area was mainly set to 5 × 5 μm^2^. The root-mean-square roughness (RMS) and height distribution range were calculated from the height profiles to quantitatively compare the nanoscale surface texture of different systems.

#### 4.4.3. μCT Scanning and Reconstruction

The internal structure of the gels was scanned using a microfocus X-ray micro-computed tomography (μCT) system (nanoVoxel-2000, Tianjin Sanying Precision Instruments Co., Ltd., Tianjin, China). The scanning voltage and current were set to 80 kV and 80 μA, respectively, with an exposure time of 500 ms per projection. A full 360° rotation was performed with an angular step of 0.5°, and the acquired projections were reconstructed using a filtered back-projection algorithm.

The voxel resolution was set to 2 μm·voxel^−1^, and a representative cubic volume of 30 × 30 × 30 μm^3^ was selected for three-dimensional reconstruction and quantitative analysis. To ensure statistical reliability, at least three independent sub-volumes were analyzed for each sample.

Pore structures were segmented using grayscale thresholding after noise reduction and beam-hardening correction. The segmented volumes were further processed by skeletonization to extract pore–throat network centerlines. Structural parameters—including average pore volume, pore volume fraction, pore–throat size distribution, node density, average connectivity path length, and connectivity ratio—were statistically derived from the reconstructed volumes using dedicated image analysis software.

#### 4.4.4. Contact Angle Measurement

Reservoir rock samples were cut into substrates of 10 mm × 10 mm × 3 mm and dried at 60 °C for 12 h. The substrates were treated as bare rock, Control-Gel-treated rock, and MPN-CNCG-treated rock. For treatment, the rock samples were immersed in 0.10 wt% solutions for 2 h, rinsed with deionized water, and dried. Static contact angles were measured at 25 °C using a JC2000D contact-angle goniometer (Shanghai Zhongchen Digital Technology Equipment Co., Ltd., Shanghai, China) by depositing 3 μL water droplets and fitting the profiles using the Young–Laplace method. Each sample was measured five times, and the average value was reported.

#### 4.4.5. Spinning Drop Tensiometry

Oil–simulated formation water interfacial tension (IFT) was measured using a TX500C spinning-drop interfacial tensiometer (Beijing Haiyida Technology Co., Ltd., Beijing, China) at different MPN-CNCG concentrations (0, 0.05, 0.10, and 0.20 wt%). The experiments were conducted at 25 °C with a rotational speed of 4000 rpm. IFT–time curves were continuously recorded over 0–180 s, and equilibrium IFT values were obtained. Each condition was tested in triplicate, and the average value was used.

#### 4.4.6. Zeta Potential Measurement

Zeta potentials of both gel dispersions and rock surface systems were measured using a Zetasizer Nano ZS zeta potential analyzer (Malvern Panalytical Instruments Co., Ltd., Shanghai, China) as a function of pH. For gel dispersions, Control-Gel and MPN-CNCG were prepared at 0.05 wt% in 1 mmol∙L^−1^ NaCl as the background electrolyte to control ionic strength. The pH was adjusted to 3, 5, 7, 9, and 11 using HCl or NaOH, and measurements were performed after equilibration at room temperature. For the rock surface system, rock powders with particle sizes < 45 μm were used to prepare suspensions. For the treated group, the rock powders were first contacted with MPN-CNCG solutions under shaking for 12 h, followed by centrifugation, washing, and redispersion in the background electrolyte solution. Zeta potentials were determined by electrophoretic light scattering. Each pH condition was measured independently three times, and the results are reported as mean ± standard deviation (SD).

#### 4.4.7. Core Flooding

Core flooding experiments were performed using a high-pressure core flooding system (HTHP-IV, Jiangsu Hai’an Petroleum Scientific Instrument Co., Ltd., Nantong, China). Cylindrical tight sandstone cores with comparable petrophysical properties were selected to minimize heterogeneity effects. The cores had a diameter of 2.5 cm and a length of 5.0 cm. Prior to experiments, core porosity and gas permeability (0.05–5.0 mD) were measured, and only cores within a narrow permeability deviation range were used for comparative tests.

Each core was mounted in a stainless-steel core holder and subjected to a constant confining pressure of 10 MPa to ensure radial sealing and simulate subsurface stress conditions. All experiments were conducted at 25 ± 1 °C. A constant injection flow rate of 0.5 mL·min^−1^ was applied throughout the flooding process unless otherwise specified. Pressure transducers installed at the inlet and outlet continuously recorded the pressure differential (ΔP, kPa) across the core with real-time data acquisition.

Injection volumes were normalized by pore volume (PV), where PV represents the effective pore volume of the core calculated from porosity and bulk volume.

The flooding procedure consisted of three sequential stages:

(i) Baseline water flooding: Simulated formation water was injected until a stable pressure differential was achieved, establishing the initial steady-state permeability (k_0_).
(ii) System injection: Control-Gel or MPN-CNCG was injected at the same flow rate, and the ΔP–PV response was continuously monitored to evaluate resistance buildup and spatial distribution behavior.
(iii) Post-flush stage: Simulated formation water was reinjected to observe pressure evolution and permeability recovery behavior.

To assess configurational stability, injection was stopped after steady-state resistance was established during system placement. After a static period of 30 min, water flooding was restarted under the same flow conditions to record pressure recovery and retention behavior during the re-flooding stage.

Each experimental condition was repeated at least three times, and freshly prepared systems were used for each test. The reported values represent averaged results.

#### 4.4.8. Steady-State Permeability

Core permeability was determined using the steady-state method based on Darcy’s law:

(1) $$k=\frac{Q\mu L}{A\Delta P}$$

where *k* is the permeability (m^2^), *Q* is the volumetric flow rate (m^3^·s^−1^), *μ* is the dynamic viscosity of the injected fluid (Pa·s), *L* is the core length (m), *A* is the cross-sectional area of the core (m^2^), Δ*P* is the measured pressure differential across the core (Pa).

During baseline water flooding, the initial steady-state permeability k_0_ was calculated once ΔP stabilized. After system injection and subsequent post-flushing, permeability values (k) were recalculated under identical flow conditions.

To eliminate geometric influence, the normalized permeability k/k_0_ was used to characterize the relative change in seepage capacity at different flooding stages.

#### 4.4.9. Oil Recovery Calculation

After completion of the core flooding experiments for both the multiscale gel–particle system and the control system, the fluids produced from each stage were collected and allowed to separate into oil and water phases. The cumulative oil production (N_p_) was determined gravimetrically after phase separation.

The recovery factor (RF) was calculated as the ratio of cumulative oil production to the original oil in place (OOIP). The OOIP was determined based on the measured pore volume (PV) of the core and the initial oil saturation established prior to flooding.

Before flooding, the pore volume of each core was measured during vacuum saturation with simulated formation water. The cores were then saturated with crude oil under controlled pressure conditions to establish the initial oil saturation (S_oi_). S_oi_ was calculated from the ratio of saturated oil volume to total pore volume. After flooding, the residual oil saturation S_or_ was calculated using a material balance method. The oil displacement efficiency *E_m_* was calculated according to Equation (2).

(2) $$E_{m}=\frac{S_{oi}-S_{or}}{S_{oi}}\times 100\%$$

Here, *S_oi_* is the initial oil saturation and *S_or_* is the residual oil saturation after displacement. The recovery factor quantifies macroscopic oil production, while the oil displacement efficiency describes the microscopic reduction in residual oil saturation. All experiments were performed under the same core physical properties and flooding conditions, and each experiment was conducted in triplicate with the reported values representing the averages.

## Figures and Tables

**Table 1:** Nanoscale surface roughness (RMS) results.

Sample	AFM Scan Area/μm^2^	Height Range/nm	RMS Roughness/nm
Control-Gel	5 × 5	0–120	23.6
MPN-CNCG	5 × 5	0–250	71.4

**Table 2:** Quantitative parameters of the μCT-derived three-dimensional structures.

Parameter	Control-Gel	MPN-CNCG
Average pore volume/μm^3^	145.3	248.7
Pore volume fraction/%	38.6	52.4
Average pore–throat size/μm	5.8	9.4
Pore–throat size distribution/μm	3–8	5–10
Node density (nodes/30 μm^3^)	336	543
Average connected path length/μm	21.4	33.1
Connectivity ratio/%	41.2	68.5

**Table 3:** Pressure differential recovery and retention characteristics during re-flooding (including recovery indices).

System	ΔP Before Stop/kPa	ΔP After Restart/kPa	ΔP After Post-Flush/kPa	Recovery Ratio R_1_ = ΔP After Restart/ΔP Before Stop	Retention Ratio R_2_ = ΔP After Post-Flush/ΔP Before Stop
Control-Gel	30.5	17.2	15.8	0.564	0.518
MPN-CNCG	19.8	18.3	17.5	0.924	0.884

Note: where R_1_ represents the pressure recovery ratio and R_2_ denotes the pressure retention ratio. Both indices are dimensionless.

**Table 4:** Oil displacement efficiency of cores treated with different systems (based on the saturation method).

System	Initial Oil Saturation, S_oi_/(−)	Residual Oil Saturation, S_or_/(−)	Oil Displacement Efficiency, E_m_/%
Control-Gel	0.72	0.46	36.1
MPN-CNCG	0.72	0.32	55.6

Note: where S_oi_ represents the initial oil saturation (dimensionless), S_or_ denotes the residual oil saturation after flooding, and E_m_ refers to the oil displacement efficiency (%). Both core groups were prepared with the same initial oil saturation (S_oi_) using an identical saturation procedure. Therefore, the key comparison lies in the magnitude of residual oil saturation reduction. These data demonstrate that MPN-CNCG significantly decreases S_or_, thereby enhancing oil displacement efficiency.

**Table 5:** Correspondence between pressure differential and recovery factor during displacement.

Injected PV	ΔP (Control-Gel)/kPa	ΔP (MPN-CNCG)/kPa	Recovery Factor (Control-Gel)/%	Recovery Factor (MPN-CNCG)/%
0.5	6.1	6.0	18.6	21.4
1.0	9.3	7.9	23.9	29.6
1.5	13.8	10.2	26.8	35.1
2.0	18.1	13.0	28.3	39.4
2.5	22.4	15.1	29.1	42.6
3.0	26.2	17.0	29.5	44.8

**Table 6:** Materials Used in This Study.

Material	Specification	Supplier	Role in System
Acrylamide (AM)	≥99.0%	Aladdin Biochemical Technology Co., Ltd., (Shanghai, China)	Main monomer
AMPS	≥98.0%	Macklin Biochemical Co., Ltd., (Shanghai, China)	Functional monomer
DMC	≥98.0%	Macklin Biochemical Co., Ltd., (Shanghai, China)	Cationic monomer
MBAA	≥99.0%	Aladdin Biochemical Technology Co., Ltd., (Shanghai, China)	Crosslinker
CNC	80–120 nm	XFNANO Materials Tech Co., Ltd., (Nanjing, China)	Nanoscale reinforcement
MPN	50–100 nm	Suzhou Nanomaterials Technology Co., Ltd., (Suzhou, China)	Nanostructured crosslink node
Polymer microspheres	30–80 μm, 20% solid	Boruite Polymer Materials Co., Ltd., (Suzhou, China)	Microscale nested unit
NaCl	≥99.5%	Xilong Scientific Co., Ltd., (Shantou, China)	Background electrolyte
HCl	0.10 mol·L^−1^	Kermel Chemical Reagent Co., Ltd., (Tianjin, China)	pH adjustment
NaOH	≥96.0%	Xilong Scientific Co., Ltd., (Shantou, China)	pH adjustment
Tight sandstone cores	0.05–5.0 mD	Xi’an Geological Testing Center, (Xi’an, China)	Core flooding medium
Crude oil	—	Shengli Oilfield, (Dongying, China)	Displacing phase

**Table 7:** Major Instruments and Testing Systems.

Instrument	Model	Manufacturer	Application
SEM	KYKY-EM6900	Beijing Zhongke Instrument Co., Ltd. (Beijing, China)	Microstructure imaging
AFM	Dimension Icon	Bruker Nano Surfaces Division (Santa Barbara, CA, USA)	Surface roughness analysis
μCT scanner	nanoVoxel-2000	Tianjin Sanying Precision Instruments Co., Ltd. (Tianjin, China)	3D structure reconstruction
Contact angle goniometer	JC2000D	Shanghai Zhongchen Digital Technology Equipment Co., Ltd. (Shanghai, China)	Wettability measurement
Spinning drop tensiometer	TX500C	Beijing Haiyida Technology Co., Ltd. (Beijing, China)	IFT measurement
Zeta potential analyzer	Zetasizer Nano ZS	Malvern Panalytical (Malvern, UK)	Surface charge measurement
Core flooding system	HTHP-IV	Jiangsu Hai’an Petroleum Scientific Instrument Co., Ltd. (Nantong, China)	Core flooding
Electronic balance	BS-210S	Sartorius (Göttingen, Germany)	Mass measurement

## Data Availability

The original contributions presented in this study are included in the article. Further inquiries can be directed to the corresponding author.
